# Living in the digital era: The impact of digital technologies on human health

**DOI:** 10.51866/ed0006

**Published:** 2022-11-30

**Authors:** Hani Salim

Since the COVID-19 pandemic, there has been an increase in the use of digital health in education, information sharing and public health surveillance.^1^ This shift is likely attributed to the widespread availability of digital technologies and devices, such as computers, multimedia technologies, smartphones and mobile applications. In this issue, we published three local articles that provide insights into the extent, benefit and risk of digital device use in young people and children.^2-4^

In terms of benefits, the use of multimedia technologies, such as animations and videos, has transformed how health information is shared with patients. In an interventional study, Siti Aisah et al. reported that the use of animated educational videos significantly increased the knowledge of anaemia prevention among adolescents compared with the control.^2^ However, stakeholders must acknowledge the consequences of digital device use, which include misinformation and impacts on health, particularly mental health. In the study conducted by Said et al. among university students, nearly half of the study population was at a high risk of smartphone addiction.^3^ On another spectrum, Nathan et al. reported a high prevalence of digital device use among children attending government pre-schools; the majority of these children did not own a smartphone but were using the devices under close supervision.^4^ More than two thirds of caregivers reported that the risk of inappropriate content exposure was the reason for close supervision in digital device use among these children.^4^

While the use of digital devices may help patients understand their illnesses better, excessive and uncontrolled use may negatively impact the overall well-being of a person, particularly young people.^5^ Better public education and awareness may be necessary to prevent digital device overuse, which could be detrimental to human health.
